# Inflammatory Breast Cancer from Metastatic Ovarian Cancer

**DOI:** 10.1155/2016/3476143

**Published:** 2016-03-07

**Authors:** Vuthinun Achariyapota, Tuenjai Chuangsuwanich, Mongkol Benjapibal

**Affiliations:** ^1^Gynecologic Oncology Unit, Department of Obstetrics and Gynecology, Faculty of Medicine Siriraj Hospital, Bangkok 10700, Thailand; ^2^Department of Pathology, Faculty of Medicine Siriraj Hospital, Bangkok 10700, Thailand

## Abstract

Metastases to the breast from tumors other than breast carcinomas are extremely rare and represent only 0.2–1.3% of all diagnosed malignant breast tumors. Furthermore, while the most common sites for advanced ovarian cancer metastases are the liver, lung, and pleura, metastasis to the breast from a primary ovarian cancer is uncommon and has only been reported in 0.03–0.6% of all breast cancers. Here we describe a case report of a 50-year-old female patient with a rare case of breast metastases from an advanced ovarian cancer, presenting as inflammatory breast cancer. Our observations emphasize the clinical importance of distinguishing between primary and metastatic breast cancer during diagnosis for the purpose of appropriate prognosis and treatment.

## 1. Introduction

Ovarian cancer is the fifth most frequent diagnosis of female malignancy. Over three-quarters of patients presented in advanced stages, III or IV. Ovarian epithelial cancers spread primarily by exfoliation of cells through the peritoneal cavity, and a minority of cases spread by lymphatic dissemination or hematogenous spread. Metastases to the breast from extramammary malignancies are rare, representing 0.2–1.3% of all malignant tumors diagnosed in the breast [1]. This report demonstrates a rare case of breast metastases from advanced ovarian cancer presenting as an inflammatory breast cancer.

## 2. Case Report

A 50-year-old woman, nulliparous, with a medical illness of hypertension, presented with discomfort in her abdomen. She had undergone left salpingooophorectomy 15 years previously because of a benign ovarian cyst.

A 15-cm tense cystic mass was palpated above the pubic symphysis. Computerized tomography (CT) revealed a cystic ovarian mass with solid nodules and septations measuring 14.7 × 13.6 × 8.8 cm, suggestive of ovarian cancer. Serum cancer markers CA125, CA19-9, and CEA were 930 U/mL, 539 U/mL, and 11.9 ng/mL, respectively.

She underwent an exploratory laparotomy surgical staging. The ovarian cyst accidentally ruptured while being removed. There was no gross residual tumor. The final pathologic reports were clear cell carcinoma of ovary without notable metastases to other organs.

After six cycles of adjuvant chemotherapy with paclitaxel 175 mg/m^2^ and carboplatin AUC6 regimen, her serum cancer markers declined until normalization. Physical examination including pelvic examination was unremarkable. Whole abdominal CT did not show residual or recurrent disease.

**Figure 1:**
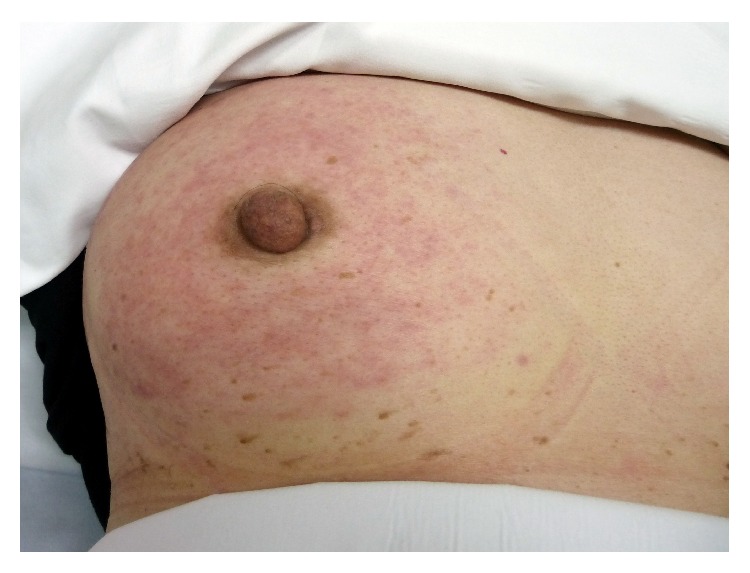
Right breast revealed red, swollen skin and peau d'orange sign.

**Figure 2:**
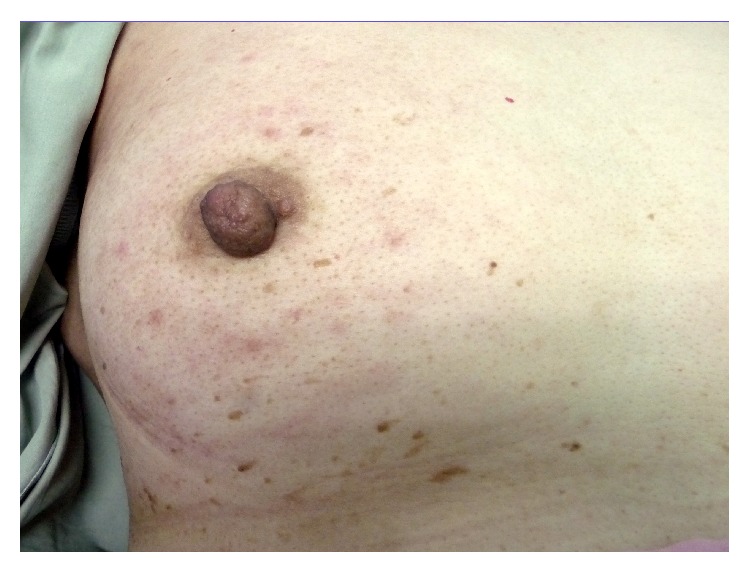
Right breast after second-line chemotherapy.

**Figure 3:**
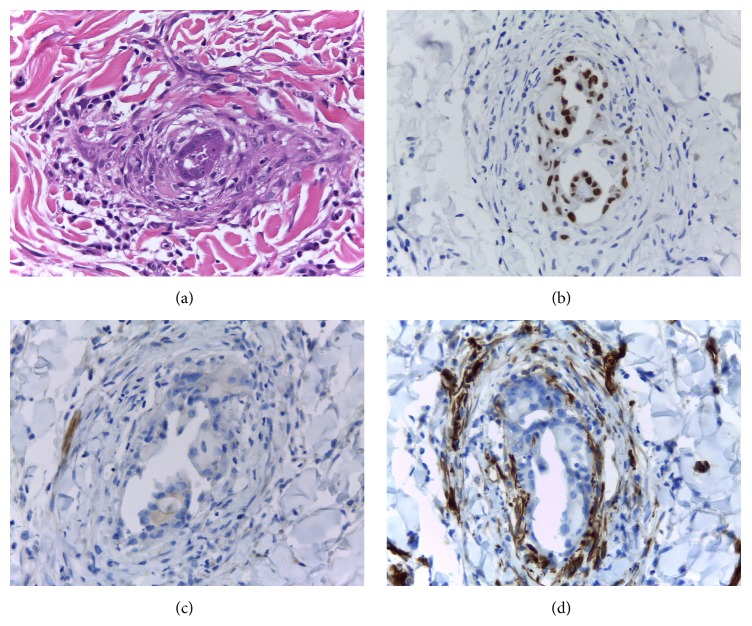
Staining of skin biopsy at right breast: (a) hematoxylin and eosin, (b) PAX8 positively, (c) GCDFP-15 negatively, and (d) WT1 negatively (×400).

Eleven months after the last treatment, she presented with erythematous patches all over her right breast, without notable nodule or ulcer. Breast examination revealed red, swollen skin and peau d'orange sign of the right breast (Figures 1 and 2), whereas the other side was normal. Mammogram revealed diffuse thickening of skin and multiple small hypoechoic nodules and benign cysts at both breasts, probably benign, BI-RADS category 3. Skin biopsy demonstrated few lymphatic spaces with tumor cells, which were negatively stained for WT-1 and TTF-1 and equivocal or negatively stained for GCDFP-15. PAX8 staining was positive in both skin biopsy and ovarian tissue (Figure 3). Her serum cancer marker CA125 was 117.20 U/mL. Metastatic adenocarcinoma was the final diagnosis. CT of chest and abdomen showed bilateral axillary and right perihilar lymphadenopathies and peritoneal metastases at the lower abdomen and perihepatic region, including some ascites and multiple enlarged intra-abdominal lymph nodes with necrotic appearance, ranging 0.6–1.1 cm in size. Retreatment was initiated and progressed through several regimens. With appropriate support in palliative care, the patient passed away 10 months later because of cancer dissemination.

## 3. Discussion

The mode of dissemination of ovarian cancer is mainly transperitoneal to the peritoneum, omentum, and bowel surface. Distant metastases are about 38% of their course of disease. Lungs, liver, and pleura are the common sites of distant metastases in advanced ovarian cancer. Metastases to the breast from extramammary cancers are rare (0.2–1.3%) [1]. Breast metastases from primary ovarian cancer have been reported in only 0.03–0.6% of all breast cancers [2]. Breast metastases presenting as inflammatory breast cancer are extremely rare, with only seven published cases [3].

Typical inflammatory breast cancer presents with rapid swelling, sometimes associated with skin changes (peau d'orange) and nipple retraction due to extensive lymphatic tumor emboli on the breast dermis [4]. In our case, tumor cells forming papillae were seen within the dermal parenchyma and lymphatic channels of the breast dermis. The mode of dissemination might originally be drainage from the intra-abdominal to axillary lymph nodes, and then the lymphatic channels of the breast.

Both primary and metastatic breast cancer can yield similar pathologic pictures and must be distinguished, because primary treatment and survival are significantly different. The most common histological variant of ovarian cancer associated with breast metastases is papillary serous adenocarcinoma. One publication demonstrated that five of seven cases that presented as inflammatory cancer were primary papillary serous adenocarcinoma of the ovary, and one case had clear cell carcinoma [3]. Clear cell carcinoma of the breast had an incidence of 0.9–2.7% [4]; thus the recognition and distinction between primary and metastases require careful consideration.

WT1, PAX8, and GCDFP-15 play a major role in distinguishing between primary and metastatic breast cancers from ovarian cancer [5], and over 90% of ovarian serous carcinomas are WT1 positive. Less than 10% of primary breast cancer cases are positive for WT1, with weak and patchy WT1 expression [6]. However, nuclear reactivity is observed in approximately 20% of clear cell carcinoma [7]. Hence, negative WT1 in this breast specimen could not distinguish primary versus metastatic breast cancer. PAX8 is positive in most ovarian nonmucinous surface epithelial tumors: serous, clear cell, and endometrioid cell types, especially for endometrioid and clear cell carcinoma, in which WT1 is generally negative or only focally positive [6]. In a study in malignant ascites, a hundred percent of primary ovarian cancers were positive in PAX8, while those of non-Müllerian origin were all negative [7]. GCDFP-15 is both highly sensitive and specific marker of breast differentiation, yielding an overall 69 and 97 percent, respectively. In a recent immunohistochemistry study, GATA3 showed superior sensitivity compared to GCDFP-15 in detecting primary breast origin, 96%. GCDFP-15 and GATA3 could be used to make a distinction between primary breast and metastatic ovarian cancer [8]. In our case, both the ovarian tumor and the tumor in the breast dermis showed similar morphology and immunohistochemistry, positive PAX8, and negative GCDFP-15, suggesting that the dermal breast lesion metastasized from the ovarian tumor as an advanced stage.

Distinguishing primary and metastatic breast cancer has an important impact in terms of prognosis and treatment. Metastatic breast cancer indicates tumor cell dissemination, which has undoubtedly poor prognosis. In ovarian cancer cases with metastasis to the breast, an average survival of 16 months (range, 13 days to 52 months) was reported [2]. Mean survival of patients with metastatic inflammatory breast cancer was even shorter, at 7 months [3]. Our patient passed away 10 months after the recurrence of diffuse metastases.

To avoid unnecessary surgical modalities, a definite diagnosis must be proven. Metastatic extramammary breast cancer has a poor prognosis and should be treated as a systemic disease with the best supportive care. The possibility of breast metastasis should be considered in patients with a history of advanced malignant disease. Definite diagnosis should be made from not only laboratory and imaging studies but also clinical presentation. When the clinical finding and morphology are obscure, immunohistochemistry study plays a major role in the differential diagnosis. A panel of WT1, PAX8, and GCDFP-15 immunoperoxidase seems to be helpful in the diagnosis.
